# Biodegradation of naphthalene, BTEX, and aliphatic hydrocarbons by *Paraburkholderia aromaticivorans* BN5 isolated from petroleum-contaminated soil

**DOI:** 10.1038/s41598-018-36165-x

**Published:** 2019-01-29

**Authors:** Yunho Lee, Yunhee Lee, Che Ok Jeon

**Affiliations:** Department of Life Science, Chung-Ang University, Seoul, 06974 Republic of Korea

## Abstract

To isolate bacteria responsible for the biodegradation of naphthalene, BTEX (benzene, toluene, ethylbenzene, and *o*-, *m*-, and *p*-xylene), and aliphatic hydrocarbons in petroleum-contaminated soil, three enrichment cultures were established using soil extract as the medium supplemented with naphthalene, BTEX, or *n*-hexadecane. Community analyses showed that *Paraburkholderia* species were predominant in naphthalene and BTEX, but relatively minor in *n*-hexadecane. *Paraburkholderia aromaticivorans* BN5 was able to degrade naphthalene and all BTEX compounds, but not *n*-hexadecane. The genome of strain BN5 harbors genes encoding 29 monooxygenases including two alkane 1-monooxygenases and 54 dioxygenases, indicating that strain BN5 has versatile metabolic capabilities, for diverse organic compounds: the ability of strain BN5 to degrade short chain aliphatic hydrocarbons was verified experimentally. The biodegradation pathways of naphthalene and BTEX compounds were bioinformatically predicted and verified experimentally through the analysis of their metabolic intermediates. Some genomic features including the encoding of the biodegradation genes on a plasmid and the low sequence homologies of biodegradation-related genes suggest that biodegradation potentials of strain BN5 may have been acquired via horizontal gene transfers and/or gene duplication, resulting in enhanced ecological fitness by enabling strain BN5 to degrade all compounds including naphthalene, BTEX, and short aliphatic hydrocarbons in contaminated soil.

## Introduction

Over the last few decades, diverse organic pollutants including petroleum fuels, pesticides, solvents, pharmaceuticals, and other organic chemicals have been produced industrially and released intentionally or unintentionally during their transports or storages^1,2^. These organic pollutants are generally highly persistent, have low degradability, and can become trapped for long periods of time in the soil minerals of contaminated sites, and subsequently bioaccumulated, due to their hydrophobic and stable chemical properties^3–5^. Because they have potentially adverse effects on human health and multiple environments (aquatic, terrestrial, and atmospheric), the contamination of these organic pollutants is a cause of great environmental concerns, which necessities the need to restore contaminated sites^1,6–10^.

Bioremediation, a technique that employs microorganisms to remove organic pollutants, has been proven to be sustainable, eco-friendly, and cost-effective among remediation technologies for contaminated sites^11–14^. Many diverse microorganisms that have good pollutant-degrading ability have been previously isolated through enrichment processes using synthetic basal media, which has led to extensive genomic and metabolic studies on pollutant biodegradation^15–20^. However, bioremediation of contaminated sites by these microorganisms often results in failure due to unfavorable several biotic and abiotic factors including low degradability, low viability, low pollutant availability, depletion of nutrients, and unfavorable pH, oxygen, temperature and moisture at contaminated sites^21–23^. Therefore, it has been suggested that the use of microorganisms mainly responsible for pollutant degradation at contaminated sites is requisite for successful bioremediation of contaminated sites^15,24–30^. Therefore, the identification and characterization of key players responsible for the degradation of organic pollutants at contaminated sites are of necessity, and enrichment cultures mimicking contaminated environments are good approaches to identify and isolate microorganisms having an ability to actively degrade organic pollutants at contaminated sites^27,31–35^.

To date, biodegradation studies have been mostly focused on the isolation and characterization of microorganisms having an ability to degrade single organic compounds although environments are generally contaminated with mixed organic compounds^33,36,37^. Therefore, a microorganism with a wide range of degradation abilities on mixed organic pollutants or a consortium of microorganisms with a degradation ability on each component of mixed organic pollutants is required for the successful bioremediation of contaminated sites. Soil of gas station is often contaminated with mixed organic compounds including BTEX (benzene, toluene, ethyl benzene, and *o-*, *m-*, *p-*xylene), polycyclic aromatic hydrocarbons (PAH), and aliphatic hydrocarbons by leaking gasoline and diesel fuels. These compounds have been recognized as environmental priority pollutants that should be removed from polluted environments because they are toxic, genotoxic, mutagenic or carcinogenic to human^6,8^. Therefore, to remediate gas station soil contaminated with gasoline and diesel fuels, microorganisms able to degrade BTEX, PAH, and aliphatic hydrocarbons may be necessary. However, bacterial members harboring degradation potentials for different types of compounds such as PAH, BTEX, and aliphatic hydrocarbons have been very rarely reported. It was reported that *Rhodococcus* strains showing broad degradation capabilities toward various compounds, including naphthalene, BTEX, aliphatic hydrocarbons were successfully isolated, but their genomic and biochemical features for their biodegradation were not investigated^38^. In this study, to identify and isolate bacteria mainly responsible for the biodegradation of BTEX, naphthalene, and aliphatic hydrocarbons from soil contaminated with gasoline and diesel fuels, we established three enrichment cultures using soil extract, supplemented with naphthalene-, BTEX-, or hexadecane, as an enrichment medium to mimic environmental conditions and isolated a bacterial strain, *Paraburkholderia aromaticivorans* BN5, which had naphthalene-, BTEX-, and short chain aliphatic hydrocarbon-biodegrading ability. In addition, in this study we investigated the genomic and biochemical features of strain BN5 for the biodegradation of naphthalene-, BTEX-, and short chain aliphatic hydrocarbons.

## Results

### Enrichment cultures and microbial community analysis

The use of microorganisms that have good pollutant-degrading ability in the contaminated site of interest, not just in the laboratory or in other sites, is a prerequisite for successful bioremediation. In this study, to isolate bacteria mainly responsible for the biodegradation of naphthalene, BTEX, and aliphatic hydrocarbons in a contaminated site, soil extract was prepared using soil from a gas station that had been contaminated with gasoline and diesel fuels for a long period of time. The contaminated soil extract, rather than a defined minimal medium, which has been commonly used previously for the enrichment of pollutant-degrading bacteria, was used as an enrichment medium.

**Figure 1:**
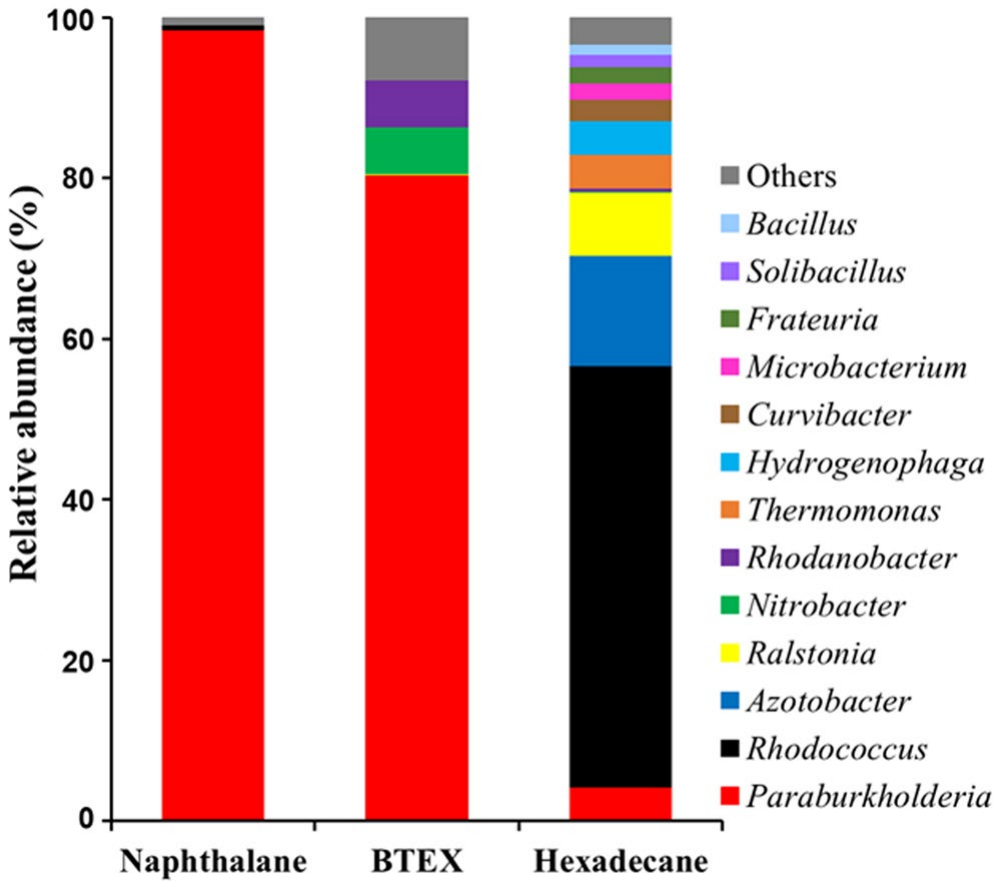
Bacterial community compositions of the final enrichment cultures supplemented with naphthalene, BTEX mixture (benzene, toluene, ethyl benzene, and *o*-, *m*-, *p*-xylene xy1:1:1:1:1:1), and hexadecane. The bacterial 16S rRNA gene sequences were classified at the genus level using the mothur software against the SILVA Gold reference database. “Others” represents taxa that comprised <1% of the total reads in three samples.

The enrichment cultures supplemented with naphthalene, BTEX mixture, and *n*-hexadecane as the sole carbon source were sub-cultured three times, and the bacterial communities of the contaminated soil used for the enrichment and the final enrichment cultures were analyzed by PacBio RS II sequencing. The bacterial community analysis showed that members of the genera *Paraburkholderia* and *Rhodococcus* were present at low relative abundances (approximately 0.9 and 0.3%, respectively) in the contaminated soil (Fig. S1). However, after the enrichments, the genus *Paraburkholderia* became predominant in both enrichment cultures supplemented with naphthalene and BTEX, accounting for 98.4% and 79.3% of their total bacterial communities, respectively (Fig. 1). On the other hand, the enrichment culture supplemented with *n*-hexadecane was dominated by the genus *Rhodococcus*, with approximately 49.8% relative abundance. The genus *Paraburkholderia* was also identified from the enrichment culture supplemented with *n*-hexadecane, but its relative abundance was only 4.0%. These data suggest that the enrichments of naphthalene, BTEX, and hexadecane degraders were successfully accomplished and the bacterial group belonging to the genus *Paraburkholderia* may degrade naphthalene, BTEX, and aliphatic hydrocarbons.

The genera *Azotobacter*, *Ralstonia*, *Nitrobacter*, *Rhodanobacter*, *Thermomonas*, *Hydrogenophaga*, and *Curvibacter* were identified from the enrichment cultures supplemented with BTEX or *n*-hexadecane as minor bacterial groups present.

### Isolation of major bacteria from the enrichment cultures and their biodegradation abilities

To isolate major bacteria with naphthalene-, BTEX-, and hexadecane-degrading abilities from the enrichment cultures, the final enrichment culture samples were spread on R2A agar and bacterial strains belonging to the genera *Paraburkholderia* and *Rhodococcus*, the dominant bacterial genus groups in the enrichment cultures, were isolated. Bacterial strains belonging to the genus *Paraburkholderia* were isolated from all three enrichment cultures supplemented with naphthalene, BTEX, and *n*-hexadecane, while bacterial strains belonging to the genus *Rhodococcus* were isolated from only the enrichment culture supplemented with *n*-hexadecane. Bacterial strains belonging to the genera *Ralstonia*, *Rhodanobacter*, and *Bacillus* that were identified as minor bacterial groups in the enrichment cultures were also successfully isolated, but the isolation of minor bacterial groups such as *Azotobacter*, *Nitrobacter*, and *Thermomonas* from the enrichment cultures failed.

The naphthalene-, BTEX-, and *n*-hexadecane-biodegrading abilities of bacterial isolates from the enrichment cultures were evaluated in sterile 160 ml serum bottles. Interestingly, all *Paraburkholderia* strains regardless of their isolation source had the ability to degrade naphthalene and all BTEX compounds, but they did not degrade *n*-hexadecane despite the fact that they were also isolated from the enrichment culture supplemented with *n*-hexadecane (data not shown). *Rhodococcus* strains isolated from the enrichment culture supplemented with *n*-hexadecane clearly showed *n*-hexadecane degradation ability, but they did not degrade naphthalene and BTEX compounds (data not shown). However, all other minor bacterial isolates did not exhibit the ability to degrade naphthalene, BTEX, or *n*-hexadecane (data not shown). Although it was reported that some *Paraburkholderia* strains may have an biodegradation ability for naphthalene or BTEX compounds, *Paraburkholderia* strains showing biodegradation abilities for naphthalene or BTEX compounds and moreover for both naphthalene and all BTEX compounds have not yet been reported. Therefore, in this study, we investigated the metabolic and genomic features of a *Paraburkholderia* strain, strain BN5, which showed the ability to biodegrade naphthalene and all BTEX compounds.

### Biodegradation of naphthalene and BTEX by strain BN5 in minimal salt basal media (MSB) and soil slurry systems

**Figure 2:**
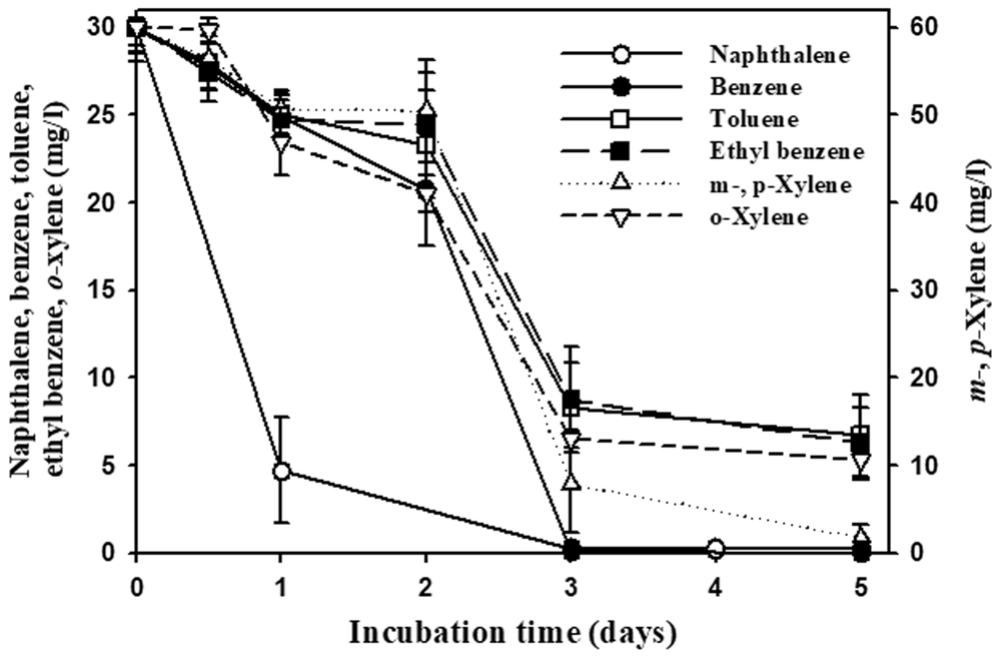
Biodegradation of naphthalene and BTEX (benzene, toluene, ethyl benzene, and *o*-, *m*-, *p*-xylene xy1:1:1:1:1:1) compounds by strain BN5 in serum bottles containing naphthalene (30 mg/l) or BTEX mixture (initial concentration of each BTEX compounds, 30 mg/l) in MSB media. Serum bottles without the inoculation of strain BN5 were used as negative controls; naphthalene and BTEX decreases in the negative controls were negligible (not shown). The symbols represent averages of triplicate experiments and the error bars indicate their corresponding standard deviations.

The ability of strain BN5 to degrade naphthalene, BTEX compounds, and *n*-hexadecane was further evaluated over time in MSB (Fig. 2). The degradation tests clearly showed that strain BN5 had the ability to degrade naphthalene as well as all six BTEX compounds. However, strain BN5 did not degrade *n*-hexadecane, even after 14 days of incubation (data not shown). Naphthalene degradation occurred more quickly than BTEX compound degradation by strain BN5, potentially because 30 mg/l of naphthalene was singly present in the test serum bottles, while 30 mg/l of each six BTEX compounds (total 180 mg/l) were present together in the test serum bottles.

**Figure 3:**
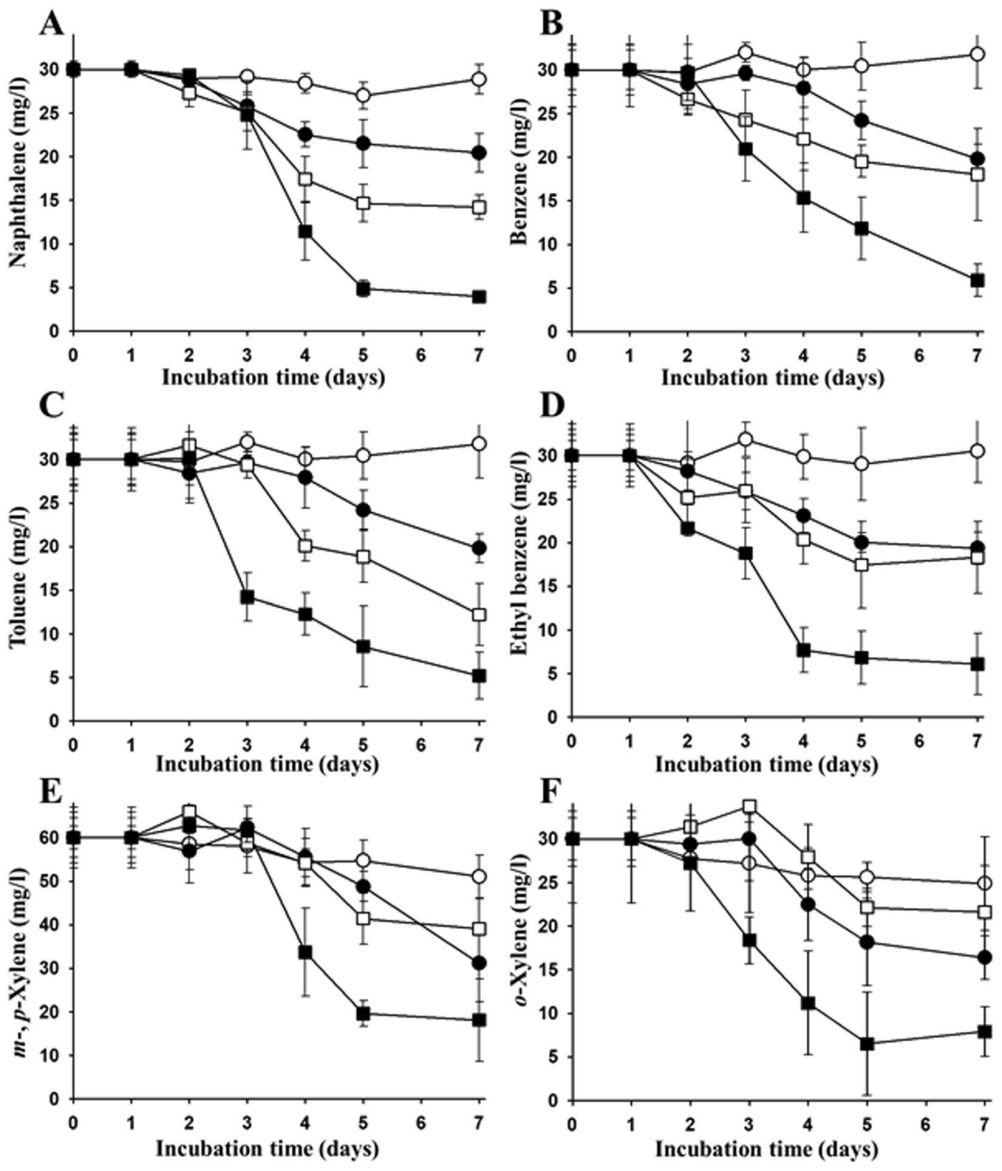
Biodegradation of naphthalene (**A**), benzene (**B**), toluene (**C**), ethyl benzene (**D**), *m*-*, p*-xylene (**E**), and *o*-xylene (**F**) by strain BN5 in soil slurry systems. Symbols in the figures are as follows: untreated (-○-), treated with nutrients (-●-), treated with strain BN5 (-□-), and treated with nutrients and strain BN5 (-■-). Unsterilized freshwater and soil were used for the setting of the soil slurry systems. The symbols represent averages of triplicate experiments and the error bars indicate their corresponding standard deviations.

The naphthalene and BTEX degradation by strain BN5 was also evaluated in soil slurry systems to better mimic the contaminated soil environment (Fig. 3). The degradation tests demonstrated that the degradation of naphthalene and BTEX compounds in the soil slurry systems did not occur, or occurred at a very low rate, without treatment with strain BN5 or nutrients (1 g/l of NH_4_Cl and 0.5 g/l of Na_3_PO_4_) during 7 days of incubation, despite the fact that the soil slurry systems were established using unsterilized soil. However, the biodegradation of naphthalene and BTEX compounds was significantly improved by the addition of nutrients. When strain BN5 was inoculated into the soil slurry systems, the biodegradation of the tested compounds was also improved. In the soil slurry systems that were treated with both nutrients and strain BN5 together, the biodegradation of naphthalene and BTEX compounds was greatly improved. These results suggest that strain BN5 was metabolically active in the degradation of naphthalene and BTEX compounds in the slurry conditions mimicking (physiologically and ecologically) the contaminated site. However, the results also suggest that there is a lack of nutrients for the biodegradation of naphthalene and BTEX compounds by bacteria in the contaminated site, and a supply of nutrients containing nitrogen and phosphorus may be necessary to improve the biodegradation.

### General features of the whole genome of strain BN5

To investigate the genomic and metabolic features of strain BN5, its whole genome was completely sequenced, and its general features summarized in Table 1. The complete genome of strains BN5 consisted of two circular chromosomes of 4,378.9 and 2,992.2 kb and six circular plasmids of 665.0, 489.4, 162.8, 152.4, 39.5, and 27.5 kb with an average G wiC content of 62.9% (Table 1 and Fig. S2). Most of the genomic features of strain BN5, such as the number of chromosomes, total chromosome sizes, total gene numbers, protein coding sequences, and total rRNA and tRNA gene numbers are relatively similar with those of other *Paraburkholderia* strains^39–41^. A phylogenetic analysis based on the 16S rRNA gene sequences indicated that strain BN5 formed a phyletic lineage within the genus *Paraburkholderia* (Fig. S3) and was most closely related to *P. phytofirmans* PsJN^T^, known to be a plant colonizing bacterium, with a very high 16S rRNA gene sequence similarity (99.4%). However, the average nucleotide identity (ANI) and *in silico* DNA-DNA hybridization (DDH) values of strains BN5 and PsJN^T^ were 88.5% and 36.5%, respectively, clearly lower than the 95% ANI and 70% DDH cut-off values generally accepted for species delineation^42,43^, suggesting that strain BN5 is a new species of the genus *Paraburkholderia*^44^. The total genome size of strain BN5 (8.9 Mb) was marginally larger than that of strain PsJN^T^ (8.2 Mb), and while strain BN5 harbors six plasmids, *P. phytofirmans* PsJN^T^ contains only one plasmid of 121 kb^40^, indicating that strain BN5 may have more diverse and versatile metabolic capabilities.

**Table 1:** General features of the chromosomes and plasmids in *P. aromaticivorans* strain BN5.

Characteristic	Chromosome (Chr) or plasmid (pBN)							
Type	Chr1	Chr2	pBN1	pBN2	pBN3	pBN4	pBN5	pBN6
Size (Kb)	4,388.9	2,992.2	665.0	489.4	162.8	152.4	39.5	27.5
G + C content (%)	63.2	63.7	61.3	60.0	59.7	61.5	59.0	59.7
Total genes	3,965	2,632	619	533	166	145	39	32
Protein coding sequences	3,823	2,535	569	483	143	137	37	29
Pseudogenes	76	81	49	50	23	8	2	2
rRNA (16S, 23S, 5S) operons	3	3	—	—	—	—	—	—
tRNA genes	54	7	1	—	—	—	—	1
Other RNA genes	4	—	—	—	—	—	—	—
Genomic islands	18	5						
CRISPRs (questionable)*	– (1)	—	—	—	—	—	—	—
Predicted transposase genes	38	28	17	52	27	9	—	—
Monooxygenase genes	14	9	1	4	1	—	—	—
Dioxygenase genes	17	23	4	10	—	—	—	—
Alkane 1-monooxygenase genes	1	1	—	—	—	—	—	—
GenBank accession numbers	NZ_CP022989–96.1							

^*^CRISPRs with fewer than three perfect repeats or nonidentical repeats are considered “questionable”.

**Figure 4:**
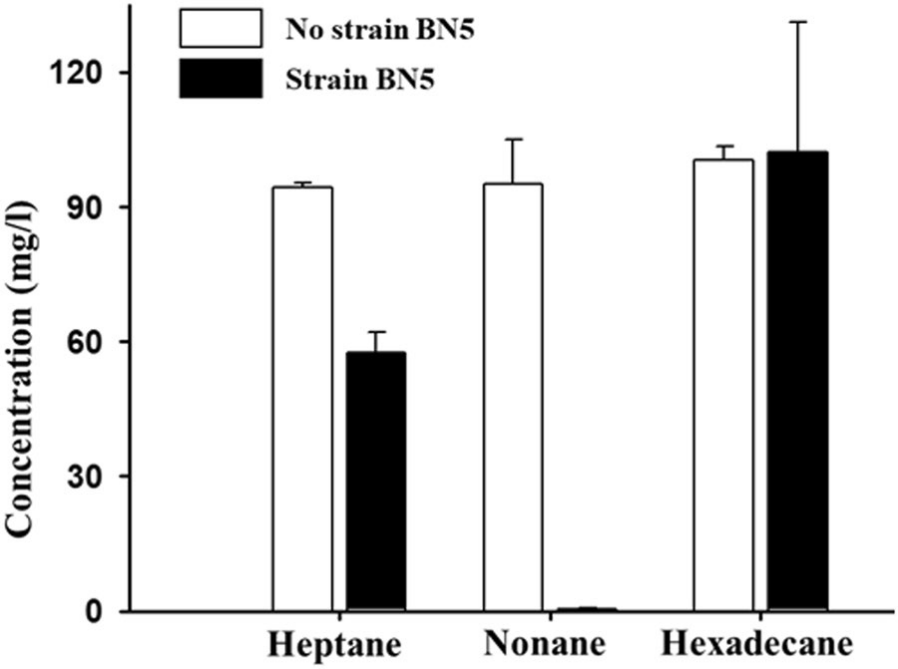
Biodegradation of heptane, nonane, and hexadecane by strain BN5 in serum bottles containing heptane, nonane or hexadecane (100 mg/l) in MSB media. Serum bottles without the inoculation of strain BN5 were used as negative controls and the concentrations of heptane, nonane, and hexadecane were measured after five days of incubation at 30 °C. The bars represent averages of triplicate experiments and the error bars indicate their corresponding standard deviations.

CRISPR, clustered regularly interspaced short palindromic repeats, along with *cas* (CRISPR-associated) genes, are a bacterial defense mechanism against bacteriophage predation and their presence has been generally accepted as an evidence for previous bacteriophage infections. Genomic analysis showed that the genome of strain BN5 contains only one possible CRISPR (Table 1), while 23 predicted genomic islands (GIs) and 171 predicted transposase genes, indicating lateral gene transfers, were identified. These findings suggests that the genome of strain BN5 has experienced extensive and complex genetic alterations or exchanges by lateral gene transfers, rather than from bacteriophage infections during its evolutionary history. Strain BN5 harbors genes encoding 29 monooxygenases and 54 dioxygenases that may be related to the biodegradation of various organic compounds including naphthalene, BTEX, and other hydrocarbons; among them, 6 monooxygenase and 14 dioxygenase genes are located on plasmids, indicating possible inflow of the biodegradation genes. Interestingly, strain BN5 also harbors chromosomal two alkane 1-monooxygenase (*alkB*) genes (CJU94_RS03555 on chromosome 1 and CJU94_RS23135 on chromosome 2). The presence of *alkB* genes suggests that strain BN5 may have the potential to degrade aliphatic hydrocarbons with different chain lengths or under specific conditions, although the strain did not show hexadecane degradation ability in the above degradation tests. Therefore, the ability of strain BN5 to degrade different aliphatic hydrocarbons with different chain lengths (heptane, nonane, and *n*-hexadecane) was evaluated. The biodegradation tests showed that strain BN5 does not degrade *n*-hexadecane, in agreement with our previous biodegradation tests, but does have the ability to degrade short chain aliphatic hydrocarbons such as heptane (C_7_) and nonane (C_9_) (Fig. 4). In particular, nonane had been completely degraded within five days of incubation.

### Genome-based prediction of naphthalene and BTEX degradation pathways and their experimental verification

Previous studies have shown that naphthalene and BTEX compounds are metabolized through a variety of degradation pathways depending on the microorganism^39^. The catabolic genes and metabolic pathways of naphthalene and BTEX compounds in strain BN5 were bioinformatically predicted and experimentally verified through gas chromatography/mass spectrometry (GC/MS) analysis of the metabolic intermediates of naphthalene and each BTEX compound. The catabolic genes of naphthalene and BTEX compounds in strain BN5 are located on plasmid 2 of 489.4 kb (pBN2), suggesting that strain BN5 probably obtained its degradation capabilities of naphthalene and BTEX compounds through plasmid transfer.

**Figure 5:**
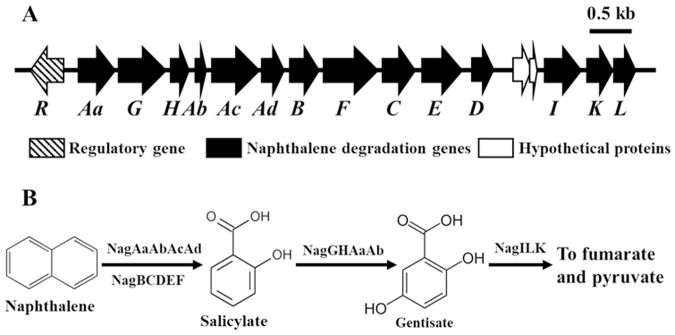
Physical map of naphthalene degradation genes located on the plasmid pBN2 of strain BN5 (**A**) and a proposed biochemical pathway of naphthalene degradation (**B**). The putative functions of the naphthalene biodegradation genes were predicted and are presented in Table S1.

It has been generally reported that one of two catabolic pathways, the catechol pathway or the gentisate pathway, is generally used by bacteria for the biodegradation of naphthalene^45^. Bioinformatic analysis of the genome showed that the naphthalene degradation gene cluster in strain BN5 is located on plasmid pBN2 and its operon structure is similar to those of *Alteromonas naphthalenivorans*^46^, *Polaromonas naphthalenivorans*^47,48^, and *Ralstonia* sp. strain U2^49^ known as the *nag* operon, involved in metabolizing naphthalene via the gentisate biochemical degradation pathway (Fig. 5A and Table S1). Figure 5A and Table S1 show the physical map of the naphthalene catabolic genes and their associated functions, respectively. Gene homology and putative functions clearly show that the naphthalene degradation gene cluster contains naphthalene dioxygenase (*nagAaAbAcAd*), salicylate-5-hydroxylase (*nagGH*), and gentisate 1,2-dioxygenase (*nagI*) genes as key naphthalene metabolic enzymes that are regulated by a LysR-type regulatory gene (*nagR*), suggesting that strain BN5 metabolizes naphthalene through salicylate and gentisate to fumarate and pyruvate, as do *Ralstonia* sp. U2^49^, *P. naphthalenivorans* CJ2^48^, and *A. naphthalenivorans* SN2^46^ (Fig. 5B). Because salicylate and gentisate are key metabolic intermediates in the gentisate pathway for naphthalene biodegradation, the presence of these intermediates was investigated using a gas chromatography-mass spectroscopy detector (GC-MSD) during naphthalene degradation by strain BN5. GC/MS analysis showed that salicylate and gentisate were detected from naphthalene-grown cell suspensions (Fig. S4), which verified that strain BN5 metabolizes naphthalene via the gentisate biochemical degradation pathway.

**Figure 6:**
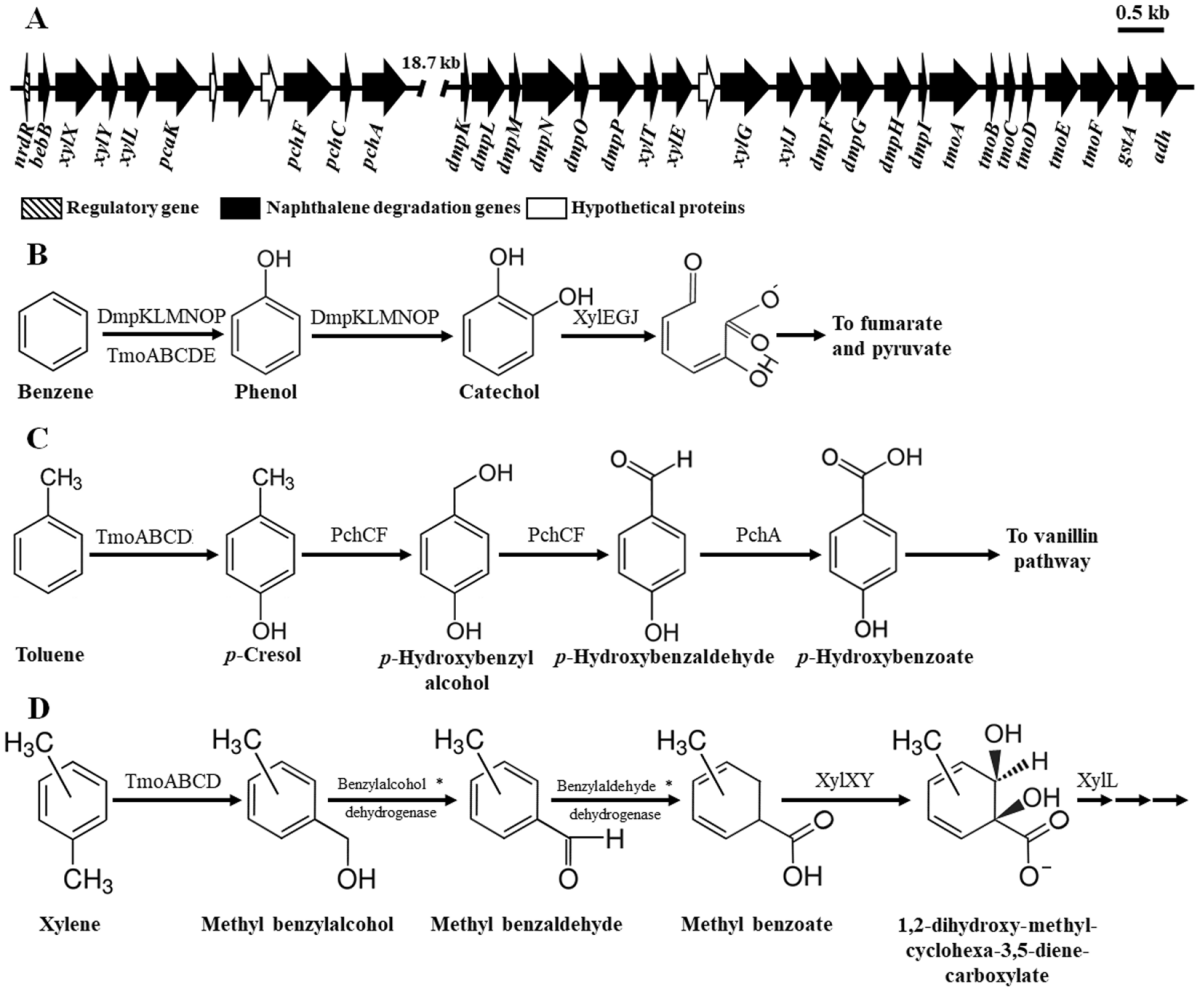
Physical maps of the metabolic genes probably responsible for benzene, toluene, and xylene degradation located on the plasmid pBN2 of strain BN5 (**A**). The metabolic genes are split into two gene clusters with a distance of approximately 18.7 kb and their putative functions were predicted and presented in Supplementary Table S2. Based on the predicted functions of the metabolic genes and the confirmation through GC/MS analysis of the metabolites, the biochemical pathways of benzene (**B**), toluene (**C**), and xylene (**D**) degradation in strain BN5 were proposed. Genes encoding the enzymes with asterisks were not identified in the genome of strain BN5 by bioinformatics analysis. The putative functions of the naphthalene biodegradation genes were predicted and presented in Table S2.

Bioinformatic analysis showed that gene clusters probably responsible for benzene/toluene/xylene degradation were also found to be located on plasmid pBN2. The benzene/toluene/xylene catabolic genes were split into two gene clusters with an approximate distance of 18.7 kb (Fig. 6A) and their associated functions are described in Table S2. Previously, two primary benzene biodegradation pathways with the conversions of benzene to phenol or benzene to *cis*-dihydrobenzenediol as the result of the first oxidation have been proposed as being typical aerobic benzene biochemical pathways^50,51^. No gene encoding benzene monooxygenase for the conversion of benzene to phenol was identified from the genome of strain BN5. However, it has been reported that toluene monooxygenase responsible for ring monooxidation of toluene and phenol 2-hydroxylase converting phenol to catechol are also able to oxidize benzene to phenol^50^. The metabolic gene cluster analysis showed that strain BN5 harbored all genes encoding toluene 4-monooxygenase (*tmoABCDE*) and phenol 2-hydroxylase (*dmpKLMNOP*) (Fig. 6A and Table S2). In addition, strain BN5 harbored genes coding for phenol 2-hydroxylase converting phenol to catechol and catechol 2,3-dioxygenase (*xylE*) converting catechol to 2-hydroxymuconate semialdehyde in the gene cluster. Based on these results, a potential benzene degradation pathway, which has phenol and catechol as intermediates, in strain BN5 was proposed (Fig. 6B). To confirm the benzene biodegradation pathway, the metabolic intermediates of benzene were investigated using a GC-MSD during benzene biodegradation: phenol and catechol were detected as metabolic intermediates from benzene-grown suspensions (Fig. S5). The detection of phenol and catechol as metabolic intermediates and the metabolic gene analysis suggest that strain BN5 metabolizes benzene through a metabolic pathway with first benzene monooxidation by toluene monooxygenase or phenol 2-hydroxylase and phenol and catechol as metabolic intermediates.

It has been proposed that aerobic toluene biodegradation is typically initiated by five different oxidations, the methyl group oxidation, ring monooxidation at position 2, 3, or 4, or ring 2,3-dioxidation of toluene, as the first step^52^. The bioinformatic analysis of the benzene/toluene/xylene catabolic genes shows that strain BN5 harbors genes encoding a complete toluene 4-monooxygenase (*tmoABCDE*), 4-cresol dehydrogenase (*pchCF*), and 4-hydroxybenzaldehyde dehydrogenase (*pchA*) as putative toluene biodegradation genes (Fig. 6A and Table S2), possibly indicating that toluene degradation in strain BN5 is initiated by ring monooxidation at the *para* position of toluene to form 4-hydroxytoluene (*p*-cresol) by TmoABCDE, followed by the conversion of 4-hydroxytoluene to 4-hydroxybenzaldehyde and 4-hydroxybenzoate by PchCF and PchA subsequently. Based on the gene annotations, a potential toluene biodegradation pathway of strain BN5 was proposed (Fig. 6C). However, the TmoABCDE- and PchCF-encoding genes were present in different gene clusters, indicating that they may be separately and independently regulated. To confirm the proposed toluene biodegradation pathway, the toluene biodegradation intermediates were investigated using a GC-MSD: 4-hydroxybenzoate was detected as a metabolic intermediate from toluene-grown cell suspensions (Fig. S6). Because 4-hydroxybenzoate is not a metabolic intermediate in four other toluene biodegradation pathways, it was concluded that strain BN5 metabolizes toluene through the toluene 4-monooxygenase pathway.

Figure 2 showed that strain BN5 has the ability to degrade all three xylene compounds, which suggests that strain BN5 harbors all xylene degradation genes. However, genes related to the xylene biodegradation, except for genes encoding benzoate 1,2-dioxygenase (XylXY) converting methyl benzoate (toluate) to 1,2-dihydroxy-methyl-cyclohexa-3,5-diene-carboxylate were not identified in the genome of strain BN5 by bioinformatic analysis. Generally, it has been reported that biodegradation of xylenes including three structural isomers of dimethyl benzene (*o*-, *m*-, and *p*-xylenes) is initiated through the oxidation of a methyl substituent of xylene to 2-methylbenzyl alcohol by xylene monooxygenase or the direct oxidation of the aromatic ring by xylene dioxygenase^53–55^. In the xylene monooxygenase pathway, 2-methylbenzyl alcohol is subsequently converted to methyl benzaldehyde, methyl benzoate, and 2-dihydroxy-methyl-cyclohexa-3,5-diene-carboxylate by benzylalcohol dehydrogenase, benzylaldehyde dehydrogenase, and benzoate 1,2-dioxygenase (XylXY), respectively. Therefore, based on the presence of XylXY-encoding genes (Fig. 6A and Table S2), a possible xylene biodegradation pathway in strain BN5 was proposed (Fig. 6D). To confirm the proposed xylene biodegradation pathway, xylene biodegradation intermediates from xylene-grown cell suspensions using each *o*-, *m*-, and *p*-xylene as a sole carbon source were investigated through GC/MS analysis. The results showed that methylbenzyl alcohol and 1,2-dihydroxy-methyl-cyclohexa-3,5-diene-carboxylate were detected as metabolic intermediates from xylene-grown cell suspensions (Fig. S7), which suggests that strain BN5 may metabolize xylene through the oxidation of a methyl substituent of xylene to 2-methylbenzyl alcohol by xylene monooxygenase. However, the genome annotation of strain BN5 failed to uncover the genes encoding xylene monooxygenase, benzylalcohol dehydrogenase, and benzylaldehyde dehydrogenase, which might be caused by their very low sequence homologies with known corresponding genes.

Two aerobic ethylbenzene metabolic pathways using either the aromatic ring oxidation of ethylbenzene by ethylbenzene dioxygenase to *cis*-1,2-dihydroxy-2,3-dihydroethylbenzene^56^ or the ethyl group oxidation of ethylbenzene by naphthalene dioxygenase to 1-phenethyl alcohol^15,57^ have been proposed. Bioinformatic analysis showed that ethylbenzene dioxygenase or genes homologous with ethylbenzene dioxygenase were not identified from in the genome of strain BN5 although it carries 29 monooxygenases and 54 dioxygenases, suggesting that strain BN5 may metabolize ethylbenzene through the ethyl group oxidation of ethylbenzene by naphthalene dioxygenase. In the ethyl group degradation pathway that uses naphthalene dioxygenase, 1-phenethyl alcohol is typically converted into 2-hydroxy acetophenone by naphthalene dioxygenase or into benzoylacetate by acetophenone carboxylase^57,58^. Therefore, metabolic intermediates of ethyl benzene biodegradation were investigated using a GC-MSD: during ethylbenzene degradation, 2-hydroxy-acetophenone was detected from ethylbenzene-grown cell suspensions (Fig. S8). Moreover, a gene encoding acetophenone carboxylase was not identified from the genome analysis of strain BN5. Based on the analysis of the genome and intermediates of strain BN5, we propose a possible ethyl benzene biodegradation pathway in strain BN5 that naphthalene dioxygenase catalyzes in multiple steps, which was in common with those in previous reports^15,57^ (Fig. S9).

## Discussion

Bioremediation using bacterial metabolic processes is considered to be one of the most efficient and eco-friendly clean-up techniques of contaminated environments^11–14^. The microbial catabolic capability for organic pollutants is a prerequisite for bioremediation practices and thus the isolation and characterization of microorganisms that have the ability to metabolize a wide range of organic pollutants has been extensively studied^11,45,53,59–61^. To date, artificial synthetic basal media containing a sole organic compound have mostly been used to enrich or isolate microorganisms that have organic substance-degradation ability on^25,26,29,31^.

However, the use of artificial synthetic basal media as an enrichment medium has often resulted in the enrichment of microorganisms that only grow successfully in laboratory conditions, not in contaminated sites^32,62^. In addition, although the isolated microorganisms have the ability to degrade organic pollutants in laboratory conditions, they often show a low degradation ability in field conditions due to various unfavorable factors^21,23,63^. Therefore, the use of microorganisms that can actively degrade organic substances at contaminated sites is necessary for the successful clean-up of contaminated sites. To identify and isolate microorganisms that effectively degrade organic pollutants at contaminated sites, various approaches such as enrichment culture^33^, metagenomics^64^, stable isotope probing^27,65^ and fluorescence *in situ* hybridization (FISH)^66^ have been used. Among them, an enrichment culture mimicking contaminated environments is one of the easiest and most promising approaches to identify and isolate microorganisms that have pollutant degradation ability at contaminated sites^33,35,67–69^. Therefore, to isolate microorganisms responsible for the degradation of BTEX, naphthalene, and aliphatic hydrocarbons in petroleum (gasoline and diesel)-contaminated soil, soil extract, not an artificial synthetic basal medium, was used as an enrichment medium.

Three enrichment cultures for the isolation of naphthalene-, BTEX-, and aliphatic hydrocarbon-degrading bacteria from gasoline and diesel fuel-contaminated soil were established. A bacterial group belonging to *Paraburkholderia* was present, with 0.9% relative abundance in the contaminated soil, and predominantly enriched in the enrichment cultures using soil extract supplemented with naphthalene and BTEX (Figs 1 and S1). The degradation tests showed that *Paraburkholderia* strain BN5 that was isolated from the enrichment cultures had the ability to degrade naphthalene as well as all BTEX compounds in a slurry system designed to mimic the contaminated soil conditions (Fig. 3), again reinforcing the idea that *Paraburkholderia* may be responsible for the biodegradation of naphthalene and BTEX compounds in the contaminated soil. However, many other biotic and abiotic factors affecting biodegradation activity of *Paraburkholderia* that we are not able to mimic in the slurry system are possibly present in contaminated soils^21,70^. In addition, it is not thought that the growth of *Paraburkholderia* with 0.9% of relative abundance in the contaminated soil entirely relies on only the biodegradation of naphthalene and BTEX compounds because contaminated soils contain many other organic carbon sources. Therefore, *in situ* biodegradation test may be necessary to assess more clearly biodegradation activity of *Paraburkholderia* in the contaminated soil.

*Paraburkholderia* species were also identified from the enrichment culture supplemented with *n*-hexadecane, although their relative abundance was low (Fig. 1) and *Paraburkholderia* strains isolated from the enrichment cultures had no degradation activity for *n*-hexadecane. However, bioinformatic analysis of the genome of *Paraburkholderia* strain BN5 showed that the strain harbors genes encoding two alkane 1-monooxygenases, a key enzyme in the aliphatic hydrocarbon biodegradation (Table 1). The biodegradation test of strain BN5 for aliphatic hydrocarbons with different chain lengths showed that strain BN5 had the ability to biodegrade short chain aliphatic hydrocarbons such as heptane and nonane (Fig. 4), which might be the reason that *Paraburkholderia* was only found in minor abundance in the hexadecane enrichment culture.

Many members belonging to the genera *Pseudomonas*, *Rhodococcus*, *Mycobacterium*, *Sphingomonas*, and *Burkholderia* have been considered to probably possess a broad range of catabolic potentials toward diverse aromatic compounds^30,39,71–73^. In particular, it has been largely assumed through the genomic analysis that many members of *Alcaligenaceae*, *Burkholderiaceae*, and *Comamonadaceae* families have biodegradation potentials towards a vast array of aromatic compounds, including several priority pollutants^39^. Auffret and colleagues reported that they successfully isolated *Rhodococcus* strains showing broad degradation capabilities toward various compounds, including naphthalene, BTEX, aliphatic hydrocarbons, and more recalcitrant compounds such as methyl *tert*-butyl ether and ethyl *tert*-butyl ether, but the genomic and biochemical features of the strains for the biodegradation of the organic compounds were not investigated^38^. This study is the first report for the enrichment, isolation, and genomic and biochemical analysis of *Paraburkholderia* with biodegradation abilities for all of naphthalene, BTEX, and short chain aliphatic hydrocarbons although there is a report that *P. phytofirmans* PsJN, a close relative of strain BN5, may have a potential degrading-activity for organic compounds^30,40,71^.

The degradation tests clearly showed that strain BN5 had a biodegradation ability for all of naphthalene, BTEX compounds, and short chain aliphatic hydrocarbons, but almost biochemical degradation gene clusters, except for the naphthalene degradation gene cluster, are not well organized (Figs 5 and 6 and Tables S1 and S2). In addition, most of these degradation genes were encoded on a plasmid (plasmid pBN2). These analyses suggest that strain BN5 may have recently acquired its biodegradation abilities via horizontal gene transfer (HGT) or the gain of a plasmid with degradation genes, resulting in successful adaptation to gasoline and diesel-contaminated soil. Phylogenetic analysis indicated that toluene monooxygenase and alkane 1-monooxygenase genes were not homologous with those of the genera *Paraburkholderia* and *Burkholderia*, indicating that they might have been introduced via HGT (Fig. S10). Although strain BN5 was able to biodegrade naphthalene, all BTEX compounds, and short chain aliphatic hydrocarbons, some key degradation genes such as xylene degradation genes were not identified in the genome of strain BN5.

In this study, we isolated *Paraburkholderia* strain BN5, capable of degrading naphthalene, BTEX, and short chain aliphatic hydrocarbons from gasoline and diesel fuel-contaminated soil. The genomic analysis of strain BN5 suggests that strain BN5 may have acquired its biodegradation capabilities via plasmid gain, HGT, and/or gene changes under the contaminated soil conditions, resulting in enhanced ecological fitness by enabling strain BN5 to degrade all of naphthalene, BTEX, and short chain aliphatic hydrocarbons. We predicted the degradation pathways of naphthalene and all BTEX compounds in strain BN5 by analyzing the naphthalene and BTEX degradation gene clusters, and the identified biochemical degradation pathways were experimentally verified through the analysis of their metabolic intermediates. The availability of strain BN5 and its complete genomic information will provide us with insights into better understanding of the physiological and genomic features of strain BN5 and its naphthalene-, BTEX-, and aliphatic hydrocarbon-degrading ability in soil environments contaminated with gasoline and diesel fuels. However, further studies on gene regulation of the biodegradation pathways in strain BN5 are necessary to understand the simultaneous biodegradation features of naphthalene-, BTEX-, and aliphatic hydrocarbons by strain BN5 in contaminated soil more clearly.

## Methods

### Soil samples and enrichment cultures

Soil extract was used as an enrichment medium to isolate bacteria responsible for degrading pollutants in a contaminated site. For the preparation of soil extract and the enrichment of naphthalene-, BTEX (benzene, toluene, ethyl benzene, and *o-*, *m-*, *p-*xylene)-, and aliphatic hydrocarbon-degrading bacteria, soil samples were obtained from a gasoline and diesel fuel-contaminated site (37°50′21.5″N 126°59′37.2″E) of a gas station located in Yangju, Gyeonggi Province, South Korea. Only freshly collected soil was used for the preparation of the soil extracts, which were prepared according to the procedure described previously^67^. In brief, 800 g of the contaminated soil was sieved through a 2 mm mesh and then mixed with 2 liters of distilled water. The mixture was vigorously agitated for 2 h at room temperature (approximately 25 °C) and centrifuged (5,000 rpm, 25 °C, 10 min). The supernatant was filtered through a 0.45 µm membrane filter (Millipore, USA) and then supplemented with NH_4_Cl (1 g/l) and Na_3_PO_4_ (0.5 g/l) for nutrients. Three cotton-plugged 500 ml Erlenmeyer flasks containing 100 ml of the soil extract and 10 g of the contaminated soil were prepared, and 0.5 g of naphthalene pellets, 1.0 ml of BTEX mixture (benzene, toluene, ethyl benzene, and *o-*, *m-*, *p-*xylene yl1:1:1:1:1:1), and 1.0 ml of *n*-hexadecane were directly added into each of three flasks. The three enrichment cultures were incubated at 25 °C with shaking (180 rpm) and subcultured (1:20) into fresh soil extract containing the same amount of each compound three times every two weeks.

### Bacterial community analysis of enriched cultures

Genomic DNA was extracted from the contaminated soil and the final enrichment cultures using a FastDNA SPIN Kit for Soil (MPBio, USA), according to the manufacturer’s instructions. The 16S rRNA genes of the genomic DNA were PCR-amplified using the barcoded universal bacterial primers 27F (5′-X-AGA GTT TGA TCM TGG CTC AG-3′) and 1492R (5′-X-TAC GGY TAC CTT GTT ACG ACT T-3′), where X denotes the 16-nucleotide long barcode. PCR conditions were as follows: 94 °C for 5 min, followed by 32 cycles of 94 °C for 45 s, 55 °C for 45 s, and 72 °C for 1 min, and 1 final cycle of 72 °C for 10 min. The PCR products were purified using a PCR purification kit (Solgent, Korea). A pooled composite was prepared by mixing equal amounts of the purified PCR products and sequenced on a PacBio RS II instrument using P6-C4 chemistry (Pacific Biosciences, USA) after SMRTbell adapter ligation at Macrogen (Korea). The PacBio raw data generated by the PacBio RS II single-molecule real-time (SMRT) sequencing were assembled in circular consensus sequences (CCSs) using the RS_ReadsOfInsert protocol in the SMRT Analysis Software (ver. 2.3) with the parameters “minFullPasses 5” and “minPredictedAccuracy 90” after removing SMRTbell adapters, and the CCSs were demultiplexed based on their PCR barcodes using the SMRT Portal^72^. The demultiplexed sequencing reads were processed using the mothur software^73^ as follows: sequences of 1,400–1,600 nucleotides with fewer than two ambiguous nucleotides were selected and chimera sequences were removed using the UCHIME program of the mothur software^74^ against the SILVA Gold reference database (ver. 123). The resulting high-quality sequences were classified using the naïve Bayesian classifier based on the SILVA reference taxonomy, with an 80% cutoff.

### Isolation of major bacteria from the enrichment cultures and biodegradation tests

To isolate major bacteria from the enrichment cultures, the final enrichment cultures were serially diluted in phosphate-buffered saline (137 mM NaCl, 2.7 mM KCl, 10 mM Na_2_HPO_4_, and 2 mM KH_2_PO_4_, pH 7.2) and spread onto R2A agar (BD, USA) and the agar plates were incubated at 25 °C. Thirty colonies from each enrichment were randomly selected and their 16S rRNA genes were PCR-amplified using the 27F and 1492R primers as described previously^75^. The PCR amplicons were double-digested with a mixture of *Hae*III and *Hha*I restriction enzymes and representative PCR products with unique restriction fragment length polymorphism (RFLP) patterns were sequenced, as described previously^75^. The resulting 16S rRNA gene sequences were compared with those of all reported type strains using the Nucleotide Similarity Search program in the EzTaxon-e server (https://www.ezbiocloud.net/)^76^.

The naphthalene-, BTEX-, and hexadecane-biodegrading abilities of the isolates with unique RFLP patterns were tested in triplicate in sterile 160 ml serum bottles containing 10 ml of minimal salt basal media (MSB)^77^ supplemented with 30 ppm of naphthalene, 180 ppm of BTEX mixture, and 100 ppm of *n*-hexadecane, respectively, as described previously^33,75^. In brief, cells of the isolates grown in R2A broth at 25 °C were inoculated into the serum bottles at a density of approximately 10^7^ cells/ml. The serum bottles were sealed with Teflon-coated rubber septa and incubated at 25 °C with shaking (180 rpm) for a week. The incubated media were extracted with methylene chloride (for BTEX and *n*-hexadecane) or ethyl acetate (for naphthalene) and the remaining BTEX, naphthalene, and *n*-hexadecane concentrations were measured using gas chromatography, as described previously^33,75^.

To infer the phylogenetic relationship of strain BN5, which shows naphthalene- and BTEX-biodegrading ability, the 16S rRNA gene sequences of BN5 and some closely related type strains were aligned using the fast secondary structure-aware Infernal aligner in the Ribosomal Database Project (RDP) (http://pyro.cme.msu.edu/spring/align.spr)^78^, and a phylogenetic tree was constructed using the neighbor-joining algorithm with the Kimura two-parameter model in the PHYLIP software^79^.

### Biodegradation of naphthalene and BTEX by strain BN5 in MSB and soil slurry systems

The biodegradation ability of strain BN5 was evaluated with naphthalene and BTEX in sterile 160 ml serum bottles containing 10 ml of MSB medium supplemented with naphthalene (30 mg/l) or BTEX mixture (180 mg/l). BN5 cells grown in R2A broth at 30 °C were inoculated at a density of approximately 2 × 10^7^ cells/ml and incubated at 30 °C with shaking (180 rpm). Three bottles per sample were sacrificed periodically and the compound concentrations were analyzed using HP 7890B gas chromatography (GC) coupled to a flame ionization detector (Agilent, USA) with an HP-5 column (30 m length, 0.32 µm inner diameter, 0.25 µm film thickness; J & W Scientific). The GC oven temperature was held at 40 °C for 0.50 min and increased at a rate of 10 °C per min to 150 °C and then at a rate of 15 °C per min to a final temperature of 260 °C, which was held for 5 min. Uninoculated serum bottles were used as negative controls.

To evaluate the biodegradation ability of strain BN5 with naphthalene and BTEX in soil slurry systems, four sets of serum bottles (untreated; treated with 1 g/l of NH_4_Cl and 0.5 g/l of Na_3_PO_4_ as nutrients; treated with strain BN5; and treated with nutrients and strain BN5) were prepared. Three grams of soil obtained from the gasoline and diesel fuel-contaminated site were transferred into 160 ml serum bottles containing 10 ml of freshwater (collected from the Han River, Korea, July 2017) (supplemented, or unsupplemented with nutrients) with naphthalene (30 mg/l) or BTEX mixture (180 mg/l). BN5 cells were inoculated into the serum bottles at approximately 2 × 10^7^ cells/ml. The serum bottles were incubated at 30 °C with shaking (180 rpm, with periodic inversion) and three bottles per experimental set were sacrificed periodically and the concentrations of naphthalene and BTEX were analyzed using GC, as described above.

### Complete genome sequencing and bioinformatic analyses

For the whole genome sequencing of strain BN5, genomic DNA was extracted using a Promega Wizard Genomic DNA purification kit (Promega, USA), according to the manufacturer’s instructions. The genome of strain BN5 was sequenced using a combination of PacBio RS II SMRT sequencing with a 10-kb library and Illumina Hiseq2500 sequencing at Macrogen (Korea). De novo assembly of the PacBio sequencing reads was performed through the hierarchical genome assembly process. Paired-end reads (101-bp) derived from Illumina sequencing were mapped on the complete genome obtained by the PacBio sequencing for error corrections. The NCBI (http://www.ncbi.nlm.nih.gov/) and Integrated Microbial Genomes (IMG; https://img.jgi.doe.gov/cgi-bin/er/main.cgi) servers were primarily used for genome analysis.

The ANI and *in silico* DDH values between BN5 and its closely related strains were calculated using a stand-alone software (http://www.ezbiocloud.net/sw/oat)^80^ and the server-based genome-to-genome distance calculator ver. 2.1 (http://ggdc.dsmz.de/distcalc2.php)^81^, respectively. Circular maps of the chromosomes and plasmids of strain BN5 were generated using the web-based CGview tool (http://stothard.afns.ualberta.ca/cgview_server/index.html)^82^. Clustered regularly interspaced short palindromic repeat (CRISPR) gene sequences were identified using an online web service (http://crispr.i2bc.paris-saclay.fr/Server/). Genomic islands (GIs) were predicted using the SIGI-HMM tool with Island Viewer 4 (http://www.pathogenomics.sfu.ca/islandviewer/)^83^.

The annotation and putative functions of genes associated with the biodegradation of naphthalene, BTEX, and aliphatic hydrocarbons were bioinformatically analyzed based on data from the NCBI genomic database, the Integrated Microbial Genomes (IMG) database, UniProt database (http://www.uniprot.org/), and BLAST searches. The catabolic pathways of naphthalene and each BTEX compound were predicted based on the annotation results of the metabolic genes.

### Biodegradation tests of strain BN5 on heptane, nonane, and hexadecane in MSB

The biodegradation of heptane, nonane, and *n*-hexadecane by strain BN5 was evaluated in sterile 160 ml serum bottles containing 10 ml of MSB medium supplemented with 100 mg/l of each of heptane, nonane, and *n*-hexadecane, as described above. BN5 cells were inoculated at a density of approximately 2 × 10^7^ cells/ml and the serum bottles were incubated at 30 °C with shaking (180 rpm) for 5 days. The concentrations of heptane, nonane, and *n*-hexadecane were analyzed using GC after methylene chloride extraction, as described above.

### GC/MS detection of naphthalene and BTEX metabolites

The catabolic pathways that were predicted based on bioinformatic analyses were confirmed through the detection of metabolic intermediates of naphthalene and BTEX compounds using a GC-MSD, as previously described^15,33^, with some modification. Triplicate serum bottles containing 10 ml of MSB media supplemented with naphthalene or each of the individual BTEX compounds (300 ppm) were prepared and incubated at 30 °C with shaking (150 rpm) for 2 days. Triplicate suspensions were combined and acidified with HCl to approximately pH 2.0 and metabolic intermediates were extracted with 10 ml of ethyl acetate. The ethyl acetate fractions were dried over anhydrous Na_2_SO_4_ and concentrated under an atmosphere of N_2_ to a volume of 500 µL. Metabolic intermediates were derivatized with 50 µL bis(trimethylsilyl) trifluoroacetamide (BSTFA) for 30 min and analyzed using a HP 7820 A gas chromatography-5977E mass spectroscopy detector (GC-MSD) (Agilent, USA) equipped with an HP-5 capillary column. The GC oven temperature was programmed to increase from 50 °C to 260 °C at 5 °C/min and then was held at 260 °C for 5 min. The peaks were identified by matching query mass spectra to those in a mass spectral reference library.

### Phylogenetic analysis of catabolic genes

Homologous amino acid sequences of naphthalene 1,2-dioxygenase large oxygenase component (NagAc), toluene-4-monooxygenase system protein A (TmoA), and alkane 1-monooxygenase (AlkB) were identified using the UniProt database and maximum-likelihood (ML) algorithm-based trees showing their phylogenetic relationships were constructed using the MEGA7 program and the tree topologies were evaluated through bootstrap tests with 1,000 iterations^84^.

## Electronic supplementary material

Biodegradation of naphthalene, BTEX, and aliphatic hydrocarbons by Paraburkholderia aromaticivorans BN5 isolated from petroleum-contaminated soil
