# Supplementary material for: Biodegradation of naphthalene, BTEX, and aliphatic hydrocarbons by Paraburkholderia aromaticivorans BN5 isolated from petroleum-contaminated soil
Source: Sci Rep. 2019 Jan 29;9:860. doi: 10.1038/s41598-018-36165-x (PMC6351602; doi:10.1038/s41598-018-36165-x)
Supplement: Supplementary file 1 — Biodegradation of naphthalene, BTEX, and aliphatic hydrocarbons by Paraburkholderia aromaticivorans BN5 isolated from petroleum-contaminated soil [file 41598_2018_36165_MOESM1_ESM.docx]

**Biodegradation of naphthalene, BTEX, and aliphatic hydrocarbons by *Paraburkholderia* *aromaticivorans* BN5 isolated from petroleum-contaminated soil**

**Yunho Lee, Yunhee Lee, and Che Ok Jeon***

Department of Life Science, Chung-Ang University, Seoul 06974, Republic of Korea

* To whom correspondence should be addressed: [cojeon@cau.ac.kr](mailto:cojeon@cau.ac.kr)

**Figure S1.** Bacterial community composition of the soil sample collected from a gasoline and diesel fuel-contaminated site. The bacterial 16S rRNA gene sequences were classified at the genus level using the mothur software against the SILVA Gold reference database. “Others” represents sum of taxa comprising < 0.5% of the total reads.

**
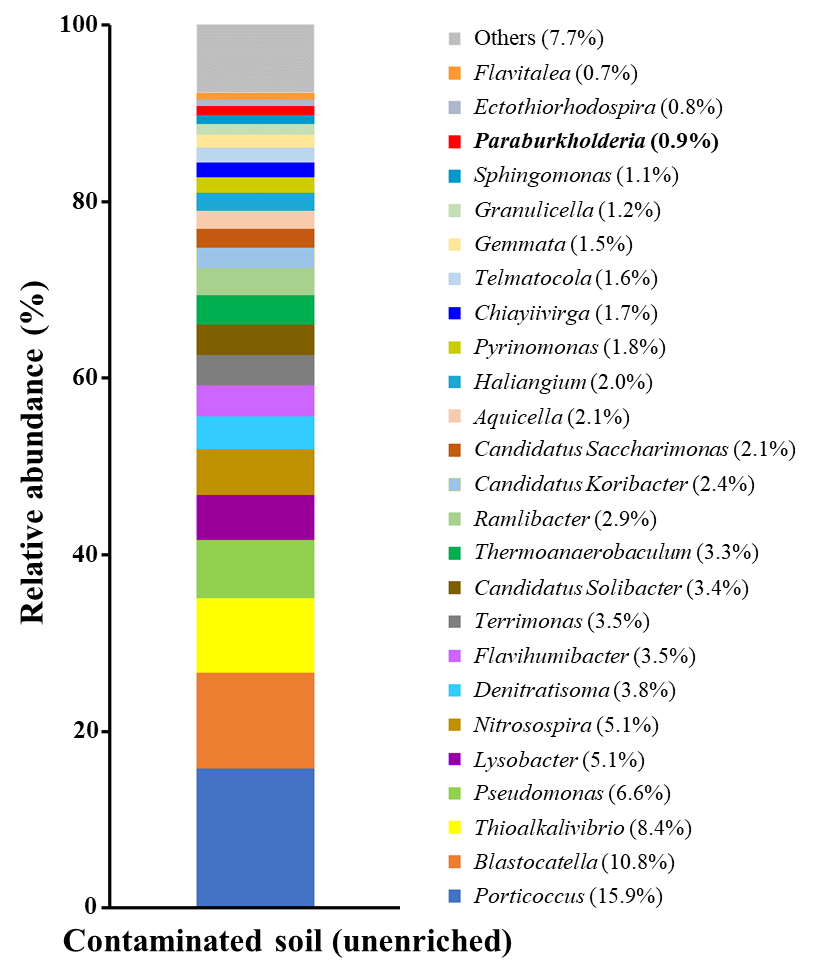
**

**Supplementary Figure S2.** Graphical maps of two chromosomes and six plasmids of *Paraburkholderia* sp. BN5 that were generated by the CGView Server. Forward- and reverse-strand protein-coding sequences are depicted on the outermost two circles (colored by COG categories) of the maps, respectively. The G+C content (black) and GC skews (positive GC skew, green; negative GC skew, magenta) are shown on the third and fourth outermost circles of the maps, respectively.

**
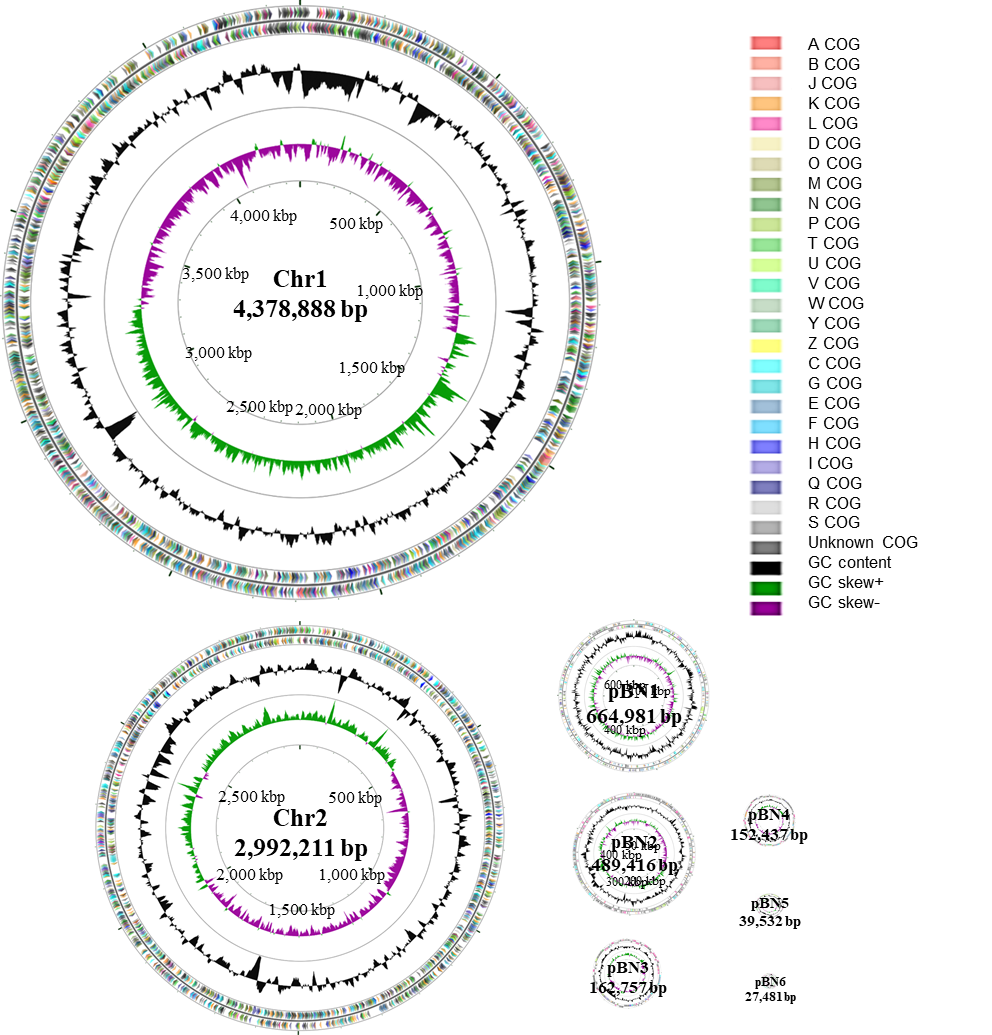
**

**Supplementary Figure S3.** A neighbor-joining tree based on 16S rRNA gene sequences showing the phylogenetic position of *Paraburkholderia aromaticivorans* BN5. Only bootstrap values above 70% are shown on the nodes as percentages of 1,000 replicates. *Cupriavidus necator* ATCC 43291^T^ was used as an outgroup. The scale bar equals 0.01 changes per nucleotide position.


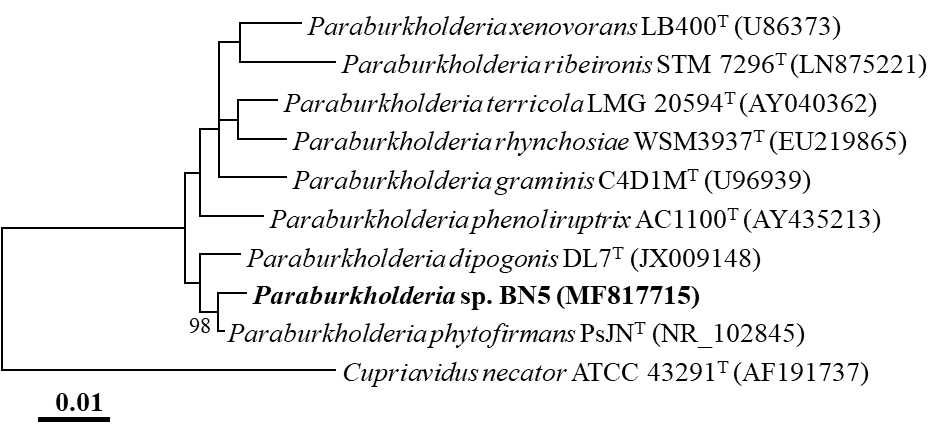


**Supplementary Figure S4.** GC/MS chromatogram (A) and mass spectra (B) of peaks documenting naphthalene metabolites, salicylate and gentisate, (analyzed as BSTFA derivatives), of strain BN5 in minimal salt basal media (MSB) supplied with 300 ppm of naphthalene.


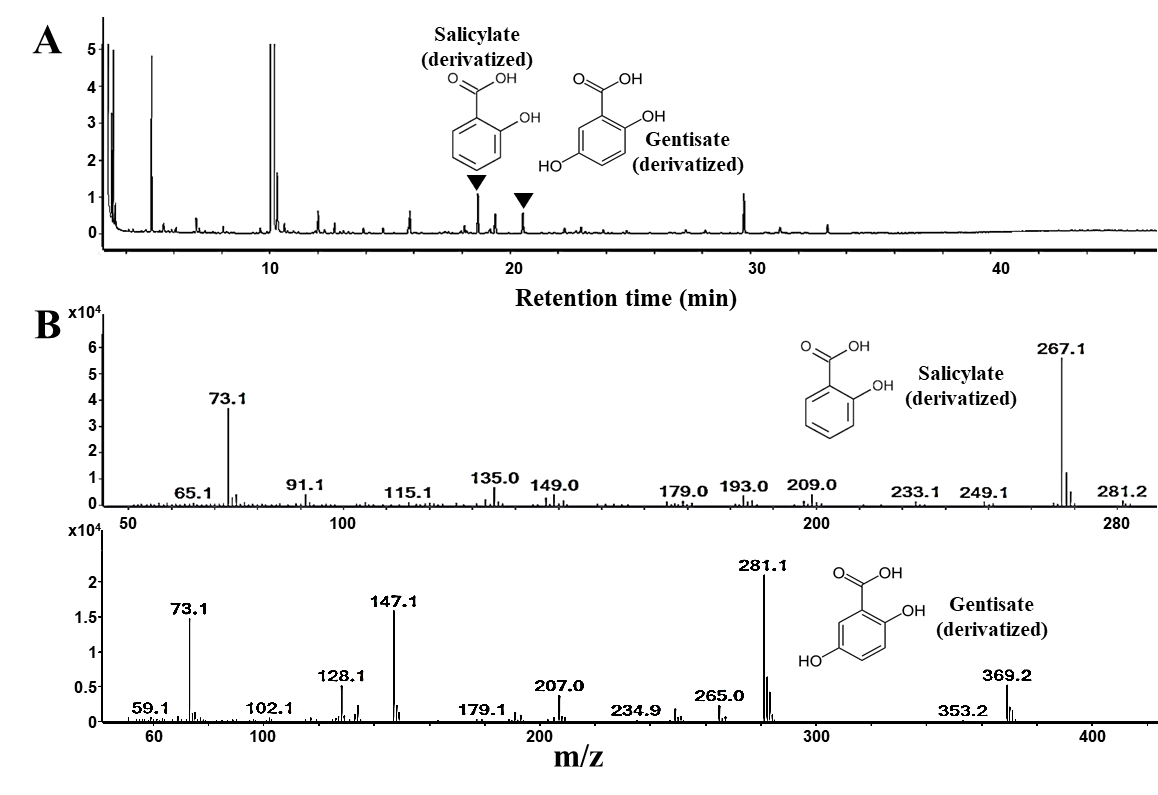


**Supplementary Figure S5.** GC/MS chromatogram (A) and mass spectra (B) of peaks documenting the benzene metabolites, phenol and catechol (analyzed as BSTFA derivatives) of strain BN5 in minimal salt basal media (MSB) supplied with 300 ppm of benzene.


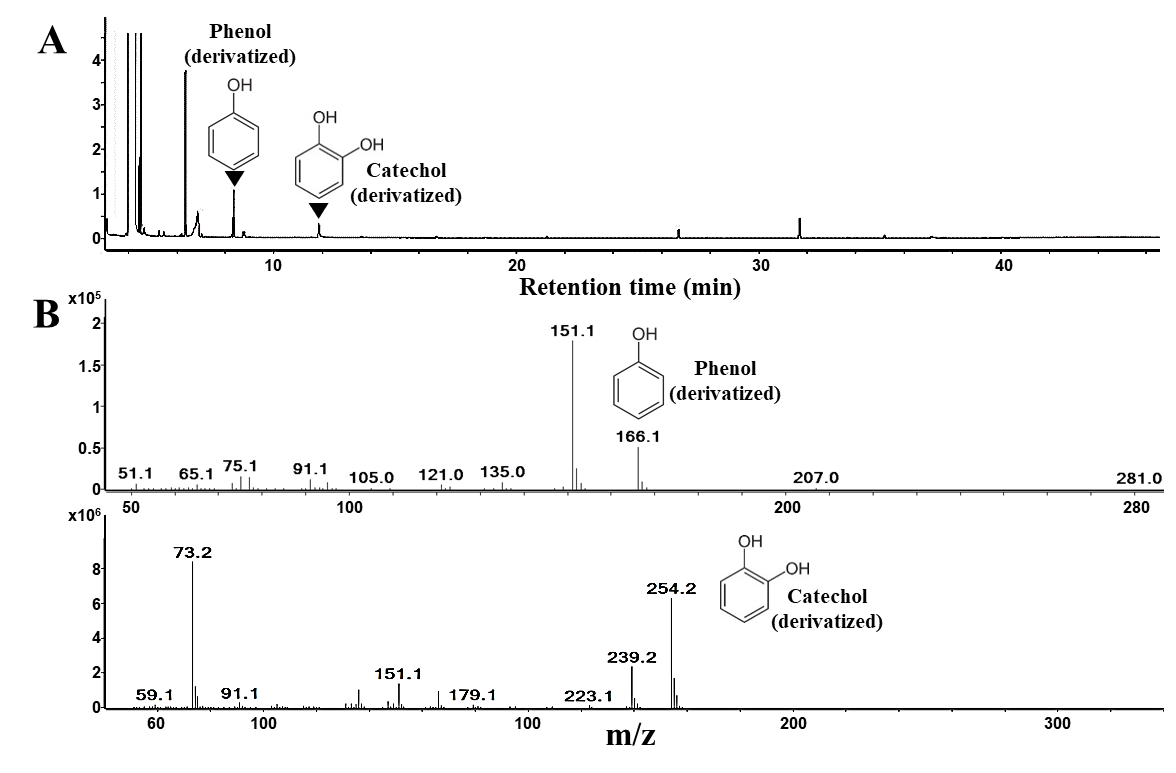


**Supplementary Figure S6.** GC/MS chromatogram (A) and the mass spectrum (B) of the peak (B) documenting a toluene metabolite, *p*-hydroxybenzoate (analyzed as a BSTFA derivative) of strain BN5 in minimal salt basal media (MSB) supplied with 300 ppm of toluene.

**
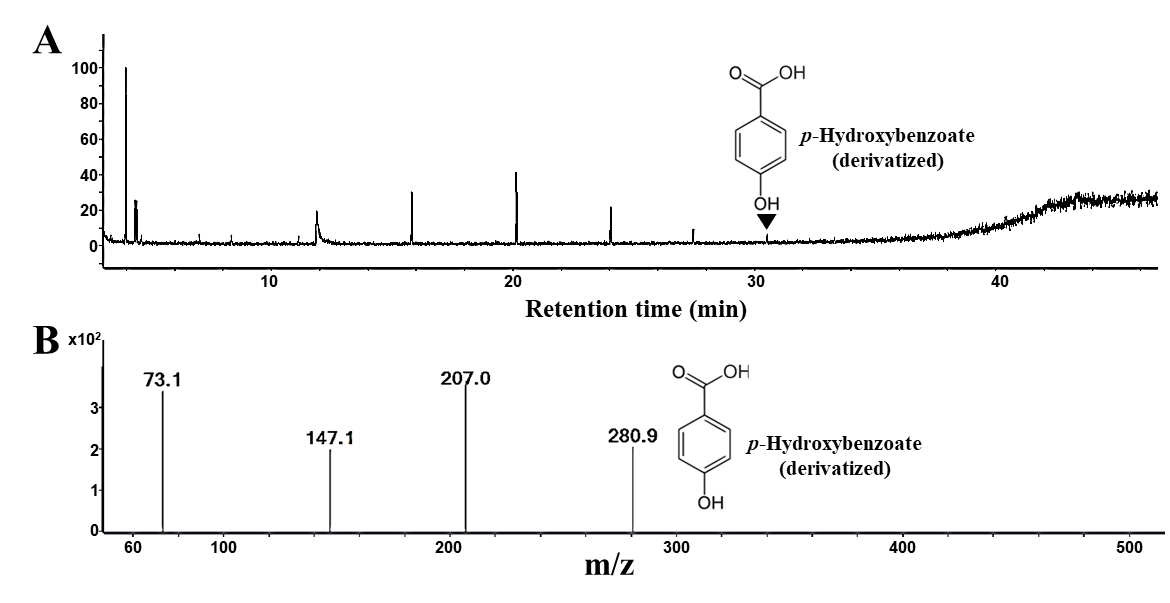
**

**Supplementary Figure S7.** GC/MS chromatograms and the mass spectra of the peaks documenting metabolites of xylene isomers (A, *o*-xylene; B, *m*-xylene; C, *p*-xylene), methylbenzyl alcohol and 1,2-dihydroxy-methyl-cyclohexa-3,5-diene-carboxylate (analyzed as BSTFA derivatives), of strain BN5 in minimal salt basal media (MSB) supplied with 300 ppm of each xylene isomer.


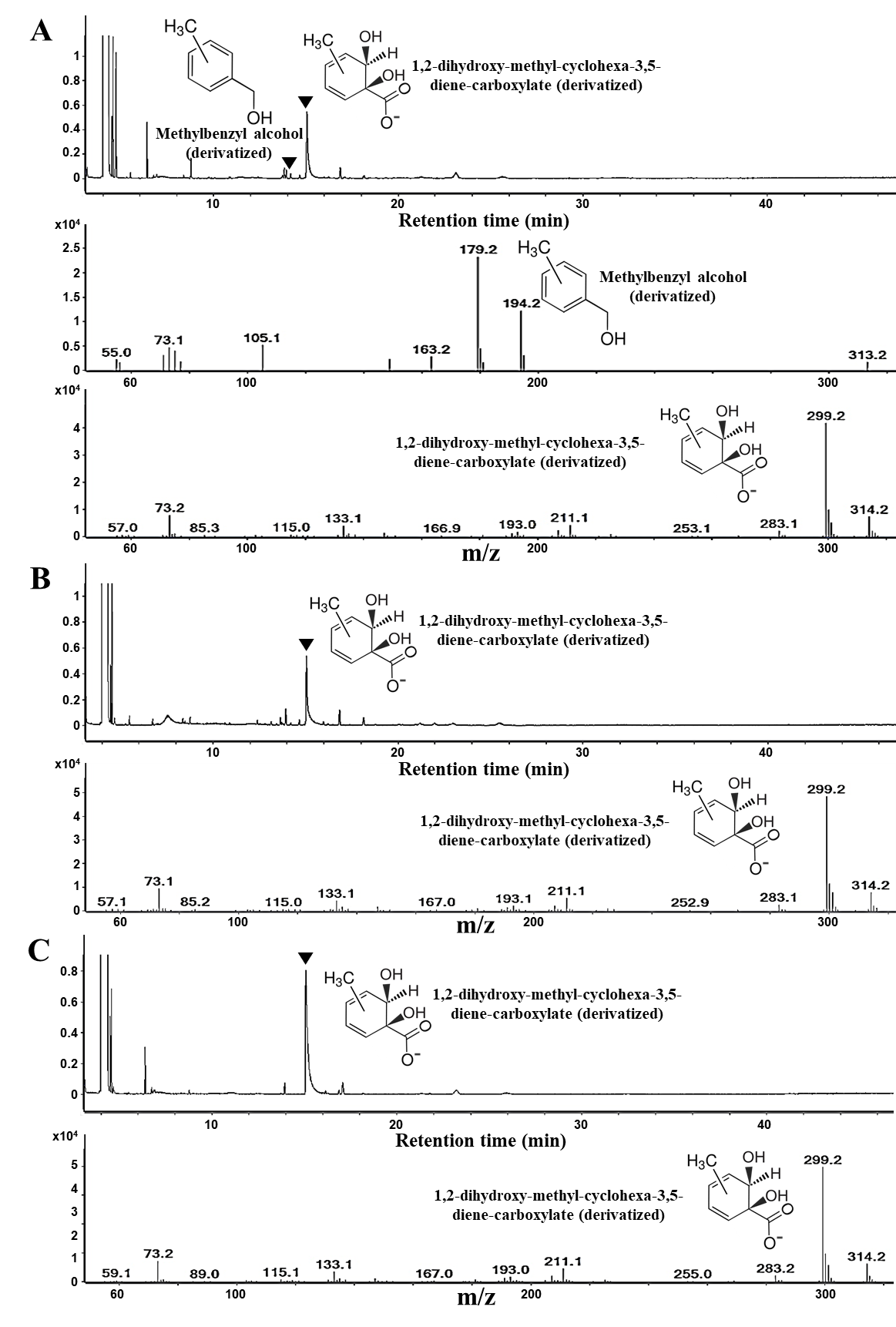


**Supplementary Figure S8.** GC/MS chromatogram (A) and the mass spectrum (B) of the peak indicating an ethylbenzene metabolite, 2-hydroxy acetophenone (analyzed as BSTFA derivatives), of strain BN5 in minimal salt basal media (MSB) supplied with 300 ppm of ethylbenzene.


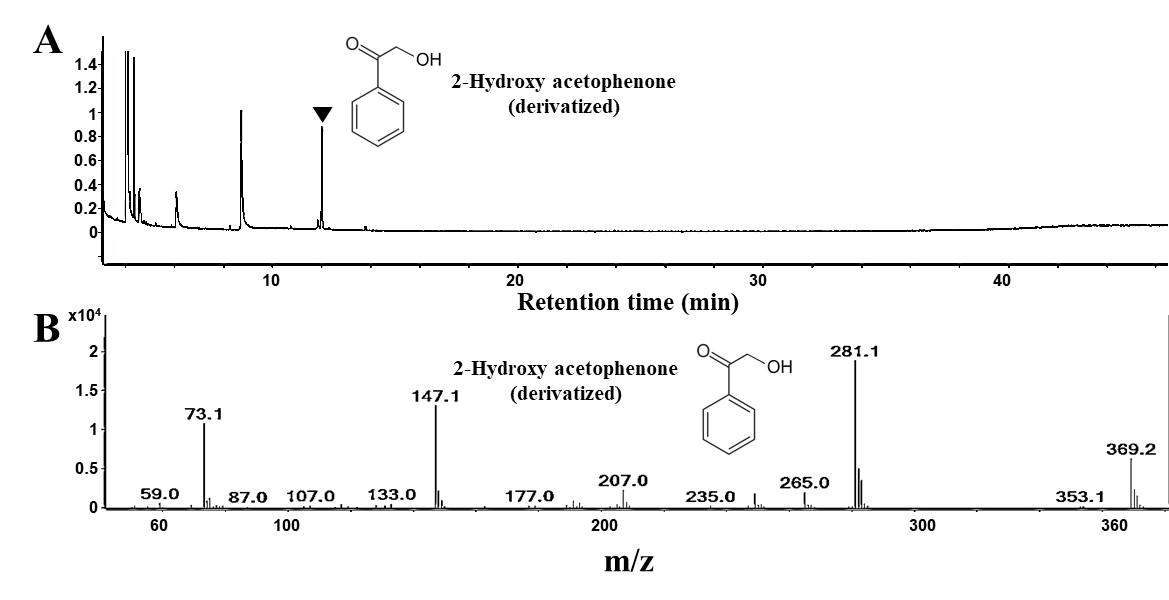


**Supplementary Figure S9.** A proposed degradation pathway of ethyl benzene in strain BN5.

**
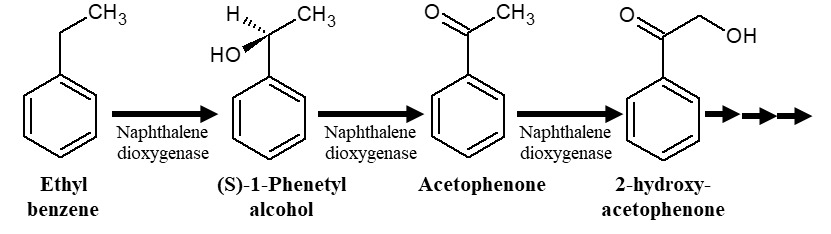
**

**Supplementary Figure S10.** Maximum-likelihood trees based on the amino acid sequences of the catabolic genes encoding naphthalene 1,2-dioxygenase (NagAc) (A), toluene-4-monooxygenase (TmoA) (B), and alkane 1-monooxygenase (C) of strain BN5 and closely related strains. Ferredoxin reductase (PhdD) of *Nocardioides* sp. KP7 (BAA94714) was used as an outgroup (not shown). The scale bars equal 0.1 changes per nucleotide position.


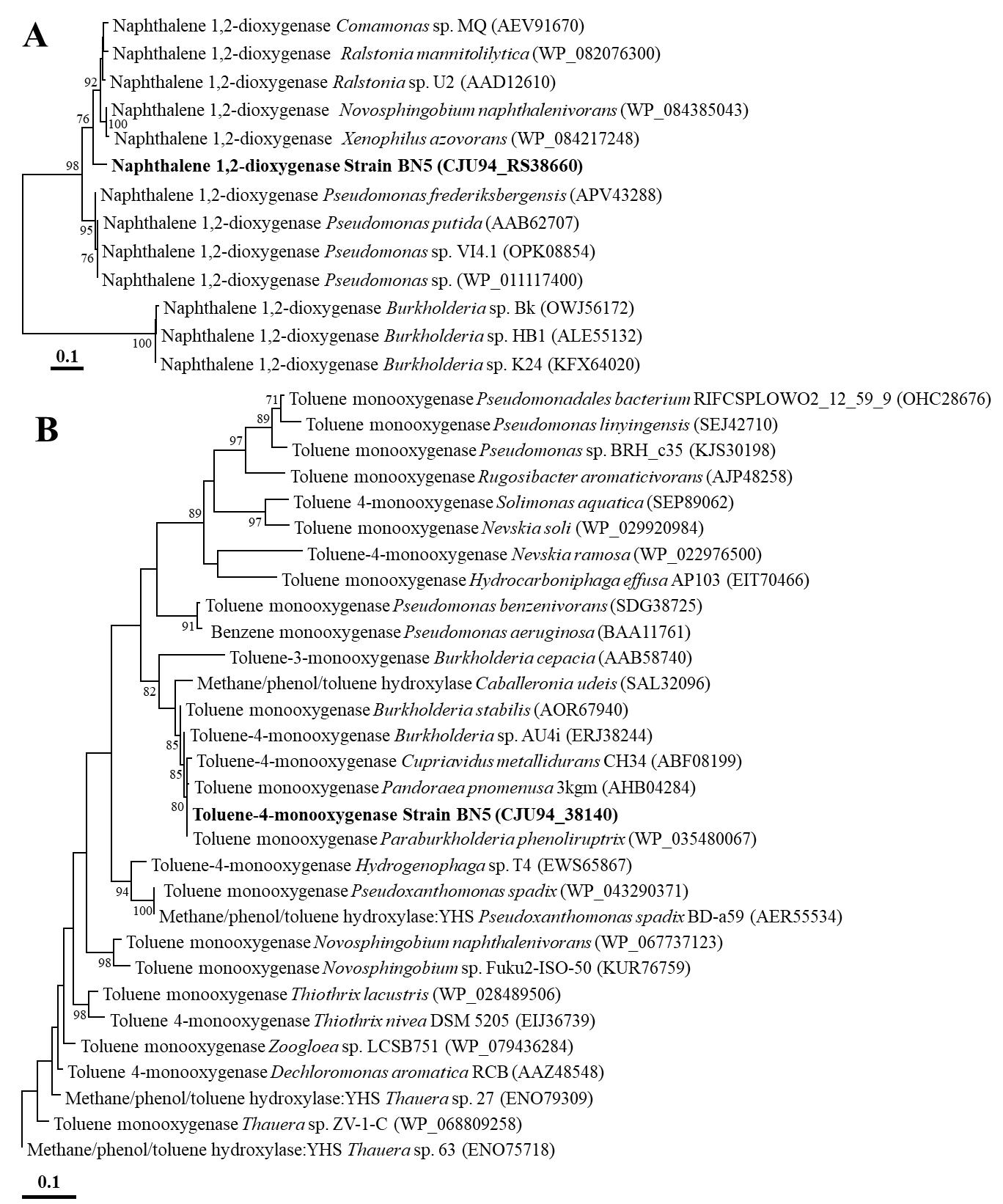


**
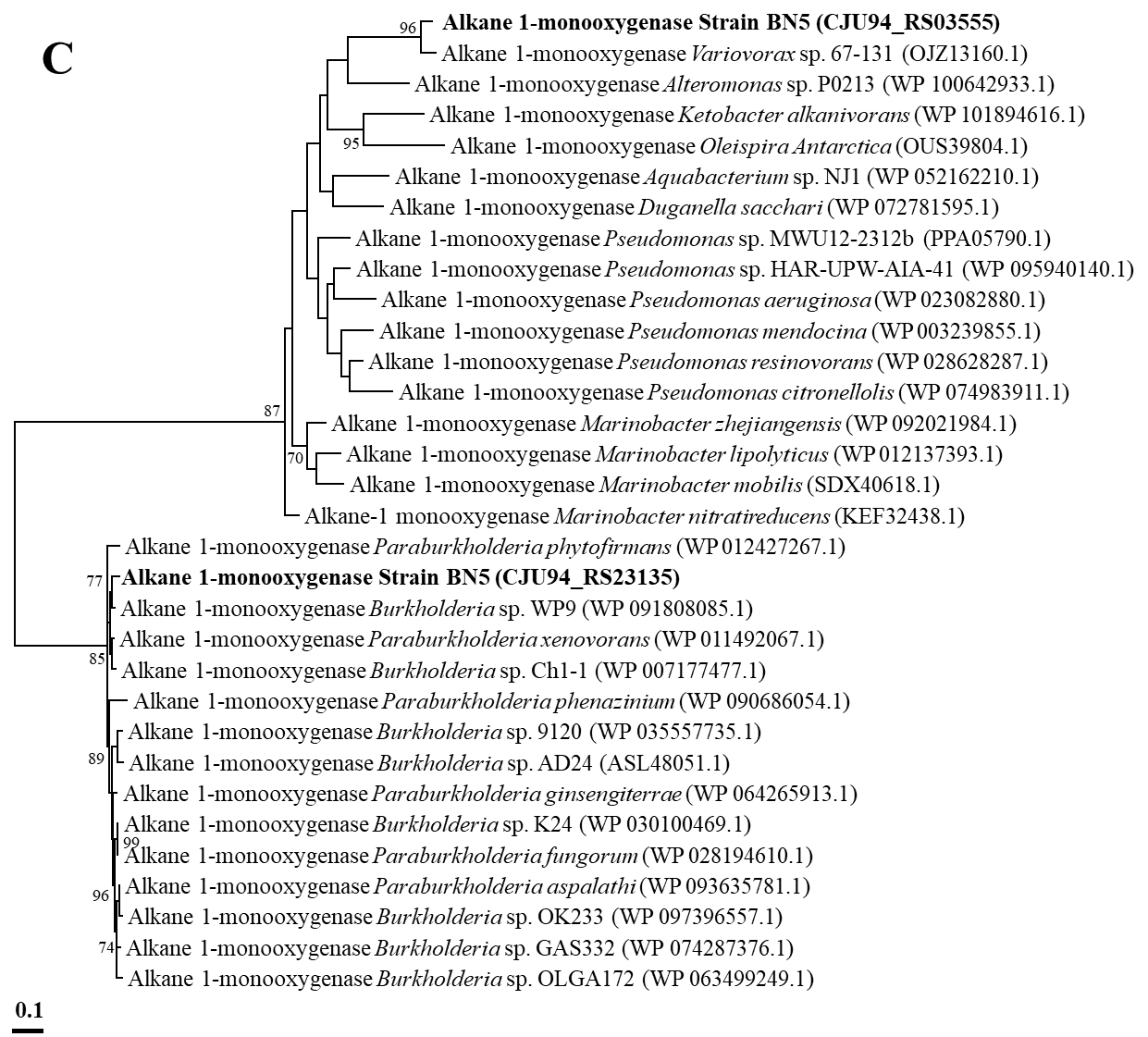
**

**Supplementary Table S1.** Naphthalene-catabolic genes encoded on the plasmid pBN2 in *P. aromaticivorans* BN5

| **Locus tag** | **Gene** | **Length (aa)*** | **Putative function** |
| --- | --- | --- | --- |
| CJU94_RS38685 | *nagR* | 315 (–) | LysR-type transcriptional regulator |
| CJU94_RS38680 | *nagAa* | 328 (+) | Ferredoxin-NAD(P)^+^ reductase |
| CJU94_RS38675 | *nagG* | 420 (+) | Salicylate 5-hydroxylase, large subunit |
| CJU94_RS38670 | *nagH* | 161 (+) | Salicylate 5-hydroxylase, small subunit |
| CJU94_RS38665 | *nagAb* | 104 (+) | Ferredoxin |
| CJU94_RS38660 | *nagAc* | 446 (+) | Naphthalene 1,2-dioxygenase, large subunit |
| CJU94_RS38655 | *nagAd* | 194 (+) | Naphthalene 1,2-dioxygenase, small subunit |
| CJU94_RS38650 | *nagB* | 259 (+) | *cis*-Naphthalene dihydrodiol dehydrogenase |
| CJU94_RS38645 | *nagF* | 483 (+) | Salicylaldehyde dehydrogenase |
| CJU94_RS38640 | *nagC* | 302 (+) | 1,2-Dihydroxynaphthalene dioxygenase |
| CJU94_RS38635 | *nagE* | 354 (+) | Trans-*o*-hydroxylbenzylidene pyruvate hydratase-aldolase |
| CJU94_RS38630 | *nagD* | 191 (+) | 2-Hydroxychromene-2-carboxylate isomerase |
| CJU94_RS38625 |  | 161 (+) | Hypothetical protein |
| CJU94_RS38620 |  | 73 (+) | Hypothetical protein |
| CJU94_RS38615 | *nagI* | 345 (+) | Gentisate 1,2-dioxygenase |
| CJU94_RS38610 | *nagK* | 236 (+) | Fumarylpyruvate hydrolase |
| CJU94_RS38605 | *nagL* | 212 (+) | Maleylpyruvate isomerase |

*aa, amino acids. The orientation of the coding strand is indicated in parentheses.

**Supplementary Table S2.** Annotation and predicted function of the benzene/toluene/xylene-catabolic genes that are encoded on plasmid pBN2 in strain BN5. The catabolic genes are split into two gene clusters, with an approximately distance of 18.7 kb, indicated by the dotted line in the table.

| **Locus tag** | **Gene** | **Length (aa)*** | **Putative function** |
| --- | --- | --- | --- |
| CJU94_RS37900 | *nrdR* | 80 (–) | Transcriptional repressor |
| CJU94_RS37905 | *bedB, todB* | 111 (+) | Ferredoxin |
| CJU94_RS37910 | *benA, xylX* | 452 (+) | Benzoate (toluate) 1,2-dioxygenase, large subunit |
| CJU94_RS37915 | *benB, xylY* | 164 (+) | Benzoate (toluate) 1,2-dioxygenase, small subunit |
| CJU94_RS37920 | *benD, xylL* | 273 (+) | 1,6-Dihydroxycyclohexa-2,4-diene-1-carboxylate dehydrogenase |
| CJU94_RS37925 | *pcaK* | 448 (+) | 4-Hydroxybenzoate transporter |
| CJU94_RS37930 |  | 67 (+) | Hypothetical protein |
| CJU94_RS37935 |  | 338 (+) | Porin |
| CJU94_RS37940 |  | 169 (+) | Hypothetical protein |
| CJU94_RS37945 | *pchF* | 524 (+) | 4-Cresol dehydrogenase, flavoprotein |
| CJU94_RS37950 | *pchC* | 111 (+) | 4-Cresol dehydrogenase, cytochrome c |
| CJU94_RS37955 | *pchA* | 482 (+) | 4-Hydroxybenzaldehyde dehydrogenase (NADP^+^) |
| CJU94_RS38065 | *dmpK* | 74 (+) | Phenol hydroxylase P0 protein |
| CJU94_RS38070 | *dmpL* | 331 (+) | Phenol hydroxylase P1 protein |
| CJU94_RS38075 | *dmpM* | 39 (+) | Phenol hydroxylase P2 protein |
| CJU94_RS38080 | *dmpN* | 519 (+) | Phenol hydroxylase P3 protein |
| CJU94_RS38085 | *dmpO, tbuD* | 118 (+) | Phenol hydroxylase P4 protein, phenol 2-monooxygenase |
| CJU94_RS38090 | *dmpP* | 354 (+) | Phenol hydroxylase P5 protein |
| CJU94_RS38095 | *xylT* | 118 (+) | Ferredoxin |
| CJU94_RS38100 | *xylE*, *bztE* | 314 (+) | Catechol 2,3-dioxygenase |
| CJU94_RS38105 |  | 149 (+) | Heme-binding protein |
| CJU94_RS38110 | *xylG*, *dmpC* | 503 (+) | 2-Hydroxymuconic semialdehyde dehydrogenase |
| CJU94_RS38115 | *xylJ* | 260 (+) | 2-Hydroxypent-2,4-dienoate hydratase |
| CJU94_RS38120 | *dmpF*, *bhpJ* | 303 (+) | Acetaldehyde dehydrogenase 2 |
| CJU94_RS38125 | *dmpG* | 348 (+) | 4-Hydroxy-2-oxovalerate aldolase |
| CJU94_RS38130 | *dmpH* | 262 (+) | 4-Oxalocrotonate decarboxylase |
| CJU94_RS38135 | *dmpI* | 63 (+) | 2-Hydroxymuconate tautomerase |
| CJU94_RS38140 | *tmoA* | 500 (+) | Toluene 4-monooxygenase, hydoxylase |
| CJU94_RS38145 | *tmoB* | 88 (+) | Toluene 4-monooxygenase, hydoxylase |
| CJU94_RS38150 | *tmoC* | 111 (+) | Ferredoxin |
| CJU94_RS38155 | *tmoD* | 104 (+) | Toluene 4-monooxygenase, effector |
| CJU94_RS38160 | *tmoE* | 328 (+) | Toluene 4-monooxygenase, hydoxylase |
| CJU94_RS38165 | *tmoF* | 340 (+) | Ferredoxin-NAD^+^ reductase |
| CJU94_RS38170 | *gstA* | 201 (+) | Glutathione S-transferase |
| CJU94_RS38175 | *adh* | 353 (+) | Alcohol dehydrogenase |

*aa, amino acids. The orientation of coding strand is indicated in parentheses.
